# A comparative assessment of major international disasters: the need for exposure assessment, systematic emergency preparedness, and lifetime health care

**DOI:** 10.1186/s12889-016-3939-3

**Published:** 2017-01-07

**Authors:** Roberto G. Lucchini, Dana Hashim, Sushma Acquilla, Angela Basanets, Pier Alberto Bertazzi, Andrey Bushmanov, Michael Crane, Denise J. Harrison, William Holden, Philip J. Landrigan, Benjamin J. Luft, Paolo Mocarelli, Nailya Mazitova, James Melius, Jacqueline M. Moline, Koji Mori, David Prezant, Joan Reibman, Dori B. Reissman, Alexander Stazharau, Ken Takahashi, Iris G. Udasin, Andrew C. Todd

**Affiliations:** 1 Icahn School of Medicine at Mount Sinai, New York, NY USA; 2 University of Brescia, Brescia, Italy; 3 Imperial College London, London, UK; 4 National Academy of Medical Sciences, Kiev, Ukraine; 5 University of Milan, Milan, Italy; 6 Federal Medical Biophysical Center, Moscow, Russia; 7 New York University, New York, USA; 8 Stony Brook University Medical Center, Stony Brook, NY USA; 9 University Milano-Bicocca, Milan, Italy; 10 New York State Laborers’ Health and Safety Trust Fund, New York, NY USA; 11 Hofstra North Shore-LIJ School of Medicine at Hofstra University, Hempstead, NY USA; 12 University of Occupational and Environmental Health, Kitakyushu, Japan; 13 New York City Fire Department, Brooklyn, NY USA; 14 New York University School of Medicine, New York, NY USA; 15 National Institute for Occupational Safety and Health, Atlanta, Georgia USA; 16 Belarus National Commission on Radiation Protection, Minsk, Belarus; 17 Robert Wood Johnson Medical Center, Piscataway, NJ USA

**Keywords:** Major accidents, Disaster epidemiology, Disaster exposure assessment, Epidemiological health surveillance, Emergency preparedness

## Abstract

**Background:**

The disasters at Seveso, Three Mile Island, Bhopal, Chernobyl, the World Trade Center (WTC) and Fukushima had historic health and economic sequelae for large populations of workers, responders and community members.

**Methods:**

Comparative data from these events were collected to derive indications for future preparedness. Information from the primary sources and a literature review addressed: i) exposure assessment; ii) exposed populations; iii) health surveillance; iv) follow-up and research outputs; v) observed physical and mental health effects; vi) treatment and benefits; and vii) outreach activities.

**Results:**

Exposure assessment was conducted in Seveso, Chernobyl and Fukushima, although none benefited from a timely or systematic strategy, yielding immediate and sequential measurements after the disaster. Identification of exposed subjects was overall underestimated. Health surveillance, treatment and follow-up research were implemented in Seveso, Chernobyl, Fukushima, and at the WTC, mostly focusing on the workers and responders, and to a lesser extent on residents. Exposure-related physical and mental health consequences were identified, indicating the need for a long-term health care of the affected populations. Fukushima has generated the largest scientific output so far, followed by the WTCHP and Chernobyl. Benefits programs and active outreach figured prominently in only the WTC Health Program. The analysis of these programs yielded the following lessons: 1) Know who was there; 2) Have public health input to the disaster response; 3) Collect health and needs data rapidly; 4) Take care of the affected; 5) Emergency preparedness; 6) Data driven, needs assessment, advocacy.

**Conclusions:**

Given the long-lasting health consequences of natural and man-made disasters, health surveillance and treatment programs are critical for management of health conditions, and emergency preparedness plans are needed to prevent or minimize the impact of future threats.

## Background

Major disasters due to industrial accidents, natural events or terrorist attacks affect workers, responders and resident populations, and can result in high numbers of deaths, injuries, physical and mental illnesses, disabilities, and home displacement. Six major accidents (summarized below) are the historic landmarks of disaster epidemiology over the last 40 years and offer crucial information on health consequences and the needs for urgent care, treatment, screening and the prevention of physical and mental health sequelae.

The disasters studied were Seveso, Italy (workers and residents exposed to dioxins [1]), Three Mile Island (reactor partial meltdown [2]), Bhopal, India (exposure to methyl isocyanate gas [3]); Chernobyl (nuclear radiation [4]), the World Trade Center (WTC) (September 11, 2001: dust cloud), and Fukushima [5] (earthquake-induced multiple reactor meltdowns resulting in radiation and displacement residents).

The aims of this study were to examine the implications of epidemiological cohort definitions and recruitment, the assessment of disaster exposure and health outcomes, and the delivery of care to affected populations after these large-scale disasters, in order to describe the health programs and interventions that were established and derive indications for emergency preparedness, focusing on event procedures and organization.

## Methods

We also covered disasters in the range of both high-income and low-income countries that span the range of established epidemiological surveillance to very little surveillance. The disasters covered were i) of major dimension and relevance and ii) with the exception of Three Mile Island, have generated programs for epidemiological health surveillance and research that our study aimed to compare. This article is unprecedented in the amount of experts from each individual disaster to weigh in and, as thus, presents a comprehensive, non-Western biased outlook on known disasters.

A PubMed literature search was performed on articles in English or with abstract in English, with no time limitations, using Medical Subject Headlines (MeSH terms) identifying each disaster. Original articles and reviewers were included by each disaster epidemiologists or experts from each respective country only if information was provided on either or including: *a*) exposure assessment; *b*) exposed populations (workers/responders and residents/survivors); *c*) health surveillance programs; *d*) research programs; *e*) physical and mental health effects; *f*) treatment and benefits; and *g*) outreach activities. For two studies in which little information had been gathered at the time of disaster, which consists of the earlier disasters of Bhopal and Chernobyl, primary data was provided by the disaster epidemiologist or expert from that particular location or country. These authors were directly involved with the health programs: (PAB, PM on Seveso; SA and DH on Bhopal; ABas, Abu, NM on Chernobyl; RGL, MC, DH, PJL, BL, JMe, JMo, DP, JR, DR, IU, ACT on WTC; KM, KT on Fukushima) for the summaries that follow. Information for Three Mile Island was available only from the literature. Lastly, after all the information was gathered and each summary written, each author reviewed the manuscript individually and discussed final strategies for the best response to large-scale disasters with the aims of exposure reduction and emergency preparedness.

## Results

### Summary description of the disasters

#### Seveso (July 10, 1976 [1])

A cloud containing 2,3,7,8-tetrachlorodibenzo-p-dioxin (TCDD) affected ~37,000 residents, causing acute chloracne skin lesions and long-term carcinogenic, cardiovascular and endocrine effects. The accident was caused by malfunction in the production of 2,4, 5 – trichlorophenol (TCP), and TCDD was formed because of high temperature generated by the runaway reaction [6]. Surface-soil-measured TCDD yielded three different areas of contamination at increasing distances from the plant: zone A (736 residents exposed to > 50 μg/m^2^); Zone B (4700 residents exposed to 5–50 μg/m^2^); and a ‘reference’ zone, ‘R’ (31,800 residents exposed to < 5 μg/m^2^). Approximately 200 children developed different types of chloracne (starting in September 1976) with spontaneous recovery [7]. Serum TCDD concentrations in approximately 3000 of the 35,000 samples collected in 1976 and 1977 were 5–3000 times higher than the background level of 10 ppt [8]. Epidemiological follow-up of 900 women showed pre-menarche TCDD exposure to be associated with a prolongation of the menstrual cycle of 0.93 days [9]; a reduced risk for leiomyoma, consistent with the anti-estrogenic activity of TCDD [10]; and no increased risk for endometriosis for serum TCDD levels less than 100 ppt [11]. A significantly lowered offspring sex ratio was shown in 450 local families, related to exposure to paternal TCDD which was approximately 20 times higher than current reference levels [12, 13] (exposure to TCDD during infancy can also reduce sperm concentration and motility [14, 15]). Excess mortality has been observed for cardiovascular and respiratory diseases, diabetes, and lymphatic and hematopoietic tissue neoplasms [16]. The Seveso Women’s Health Study has shown elevated rates for all cancers combined after 30 years [17]. Although the health surveillance program is no longer active, follow up research is still ongoing. The Seveso accident prompted better legislative control of industrial emission *via* the EU “Seveso” Directive 82/501/EEC of 1982 [18].

#### Three Mile Island (March 28, 1979)

After two nuclear accidents in military installations in Kyshtym (Russia [then USSR], 1957) and Windscale Piles (UK, 1957), the first major civilian nuclear accident occurred at the Three Mile Island Nuclear Generating station in Dauphin County near Harrisburg, Pennsylvania. Defined as a partial nuclear meltdown, it was due to critical human factors and problems in the control system’s user interface. Although evacuation of pregnant women and preschool children was extended to 20 miles radius of the facility, more than a half of the 663,500 population remained in the area and 98% of the evacuees returned to their homes within 3 weeks [19]. No effective plan was in place for hospital and nursing care facility evacuation [20]. The Maximum effective dose was 40 mSv for the emergency worker) and 0 · 015 mSv (average); 0 · 85 mSv (maximum) for the residents living within 80 km radius [21]. These levels were lower compared to those measured for the later nuclear accidents of Chernobyl and Fukushima. Small elevation was noted in risk for cancer of the bronchus, trachea, and lung, and for leukemia [2]. Although thyroid cancer incidence is greater than expected in the counties analyzed when compared to local and national population growth, a direct correlation to the accident remains uncertain [22]. Studies by the Behavioral Taskforce of the President’s Commission [23] concluded that the most relevant public health effect was on mental health, including symptoms of psychological distress in mothers of young children and emergency workers [24].

#### Chernobyl (April, 26, 1986 [4])

Evacuation of Pripyat, the city closest to the nuclear power plant (NPP) was ordered within hours; 10-km-radius evacuation the next day; and 30-km-radius evacuation on May 2^nd^. In all, 164,700 residents were ordered to evacuate.

Workers (176) and firefighters (250) were at the NPP immediately after the accident and a total of 600 were acutely exposed to high doses of radiation (whole-body γ, skin γ and β, ανδ vapor-phase radionuclide inhalation); the products of melting and burning graphite, bitumen and plastics; dust and debris from building collapses; and the sulfate-spirit bards, hydrochloric acid, formaldehyde, and oxalic acid from tens of thousands of tons of paper waste used for dust suppression. Polymeric foaming compositions based on urea resins and polyvinyl dispersed mixture were also used in large amounts for dust suppression. During 1986–1987 approximately 230,000 civil and military personnel worked as “liquidators”, cleaning up fallout within the 30 km radius. The number of liquidators ultimately reached 600,000 and they received an average absorbed dose of 9 cGy in 1987 and 5 cGy during 1988–1990.

Annual monitoring and specialized treatment for liquidators and residents are conducted in six regional centers and in specialized departments in 89 regions of the Russian Federation. Exposure data are collected into the Russian State Medical Radiation Monitoring Registry (RSMRMR) and, for health outcomes, the National Radiation and Epidemiological Registry (NRER: implemented and linked to the RSMRMR in 1993 and linked to registries in Belarus and Ukraine). As of January 1, 2016, a cohort of 762,721 individuals is registered in the NRER, including liquidators, residents and their children. Although not implemented at the time of the accident, these registries have monitored health status, yielding prediction-model-based physical and mental health treatment programs and the assessment of the relationship between the radiation exposure and the stochastic effects thereof (*e.g.*, leukemia, solid tumors and thyroid cancer).

The Russian liquidators suffered increased leukemia incidence (145 cases) which peaked from 1992 to 1995 and which was similar to the incidence rate among the atomic bomb survivors. The excess risk for leukemia decreased ~9% per year after 1995, approaching the ‘background’ Russian level in 2012 [25]. The registries in Belarus and Ukraine showed a similar trend. Thyroid cancer also increased among the liquidators [26] but the highest incidence was observed among children residing in the Bryansk region of Russia which was affected by radiation-contaminated sediment [27]. A retrospective study of cancer incidence among the emergency workers from 1992 to 2009 showed a statistically significant elevation in cancer incidence rate, but not in mortality from all cancers, relative to the total cancer incidence rate for men in Russia [28].

The State Register of Ukraine, established in 1991, includes 2,646,106 residents, as of January 1, 2014, which is estimated to be 93% of the people exposed. Medical surveillance from 1987 to 2005 recorded 504,117 deaths (497,348 adults and adolescents – 34,499 of whom were emergency workers – and 6769 children [29]). Emergency worker mortality from tumors was three times higher than in similar age groups in the general population in 2007 [29].

The Belorussian National Register (825,703 subjects) and the Joint Chernobyl Register of Russia and Belarus (309,000 subjects), were established in 1993 and are still active. Belarus annually surveys 1,527,188 people, of which 54,468 are liquidators. New medical facilities, institutes, specialized clinics and centers were opened in Belarus for this program. Research and medical follow-up showed, in 1996, an elevated incidence of thyroid cancer among subjects exposed to iodine radionuclides during childhood and adolescence [30, 31]. In a study involving children who lived within the Gomel region near Chernobyl, 244 of the 251 children born between 1986 and 1993 developed thyroid cancer [32]. The papillary thyroid cancer incidence among the 99,693 liquidators in the National Register is currently comparable to the average national level and does not appear to be increasing in either men or women.

#### Bhopal (December 2, 1984 [3])

In Bhopal, more than 500,000 workers and residents were exposed to methyl isocyanate gas and other toxins [33]. Fatalities were seen immediately with bodies and animal corpses piling around the area within hours. Hospitals were overwhelmed and neither reinforcements nor epidemiological expertise were available. Estimates of the number of people killed in the first few days by the plume from the plant range up to 10,000, and 15,000–20,000 premature deaths have reportedly occurred in the subsequent two decades [34]. Immediate primary causes of death suggest effects of severe toxicity: choking, reflexogenic circulatory collapse, and pulmonary edema, cerebral edema, tubular necrosis of the kidneys and fatty degeneration of the liver and necrotizing enteritis. In addition, spontaneous abortions occurred in approximately half of the women in their first trimester at the time [35]: the stillbirth rate tripled and neonatal mortality doubled [36].

Early reports of health effects were descriptive and no immediate exposure assessment was accomplished due to lack of planning, organization, and expertise. The knowledge of the long-term health effects of the exposure has also been limited by the absence of a structured longitudinal health surveillance program. A high proportion of the exposed population fled when the plant was restarted to burn the residual methyl isocyanate, without returning. This exodus resulted in a major limitation for the implementation of epidemiological health surveillance. Two years after the incident, the Indian Council of Medical Research (ICMR) established a cohort of civilians and workers affected by the disaster [37] but operational problems included inadequate staffing and equipment (20 research assistants to monitor a cohort of more than 95,000 persons) [38]. The ICMR ceased follow-up before 1993 and concluded its investigations by 1994. The Centre for Rehabilitation Studies of the Bhopal Gas Tragedy Relief and Rehabilitation Department – under the administrative control of the Madhya Pradesh state government – subsequently took over, restarting epidemiological assessment in 1996. Neither research group was able to collect data on maternal health or mental health services. Furthermore, local mental health services were inadequate with regard to the provision of long-term mental health care to all who needed it, and did not link of primary health care with mental health care. Public mental health education to prevent adverse long-term health consequences, self-care instructions and psycho-social interventions were also all inadequate. In addition, poor coordination with voluntary organizations resulted in mistrust and many residents moved. By 2010, the researchers had lost 79% of the cohort [39] and in 2011, the cohort was handed back to ICMR and the National Institute for Research in Environmental Health (NIREH) in Bhopal.

#### World Trade Center (WTC, September 11^th^, 2001–9/11)

In addition to the 2966 killed (2.606 in the WTC and surrounding areas, 125 at the Pentagon and 265 on the four planes), an estimated number of 90,000 workers and responders from every US state, and ~400,000 residents/vicinity-workers (hereafter ‘survivors’, *per* their Health Program name),, were affected by the events at the WTC [40]. The Fire Department of New York (FDNY) soon after the attack reported respiratory effects in firefighters and emergency medical technicians (EMTs) and Mount Sinai and collaborating occupational health centers worked on a proposal to the federal government to provide screening exams for the non-firefighter (hereafter ‘general’) responders who started presenting within a few days. The WTC Worker and Volunteer Medical Screening Program was established with funding provided by the Centers for Disease Control and Prevention (CDC)/National Institute for Occupational Safety and Health (NIOSH) and the first screening examinations occurred in July 2002. The National Institute for Environmental Health Sciences (NIEHS) also provided initial funding for research. A similar program had been established by the CDC for New York City firefighters in 2001–2002. General responders who provided rescue, recovery, demolition, debris cleanup or restoration of services received a one-time medical evaluation. Physical and mental health treatment services were provided with support from philanthropic sources. In 2004, NIOSH provided funding for additional initial medical evaluations and monitoring examinations every 18 months for what became the General Responder Cohort (GRC), and continued funding for the FDNY to provide the same services to the firefighters. In 2006, NIOSH provided funding for treatment of both physical and mental health conditions, renaming the program the WTC Medical Monitoring and Treatment Program (MMTP). Local community and academic efforts resulted in the first organized surveillance program for community members with an initial CDC-funded study of the local residents by New York University and Bellevue Hospital working with the New York State Department of Health [41, 42]. This was followed by the development of the New York City Department of Health and Mental Hygiene (NYCDOHMH) WTC Health Registry (WTCHR) [43]. In contrast to the WTC MMTP, the community programs did not provide health surveillance and clinical examinations. Small programs (initially self-funded pilot programs that received support from philanthropic sources in 2005, the City of New York in 2006, and the federal government in 2008) were developed for the treatment of WTC-related illnesses in the local community. With the passing of the James Zadroga 9/11 Health and Compensation Act of 2010, the WTC Health Program (WTCHP) was established which provided five more years of medical monitoring and treatment for both responders and the community, until July 2016.

The WTCHP provides medical monitoring and treatment for health conditions listed at www.cdc.gov/wtc/conditions.html that have been recognized as 9/11-related: i) Aero-digestive Disorders; ii) Mental Health Conditions; iii) Musculoskeletal Disorders; iv) Cancers (www.cdc.gov/wtc/coveredcancers.html).

Periodic health assessments provided to the GRC are standardized and include a medical questionnaire on WTC-related symptoms and conditions; a one-time exposure assessment questionnaire to capture exposures related to 9/11 and occupational exposures; and physician examination. Because the main exposure route for the caustic WTC dust was *via* inhalation, pulmonary function tests (PFTs) are conducted and chest x-rays are taken. Routine blood work and urine analysis are also included [44]. In addition, a self-administered mental health screening questionnaire identifies general psychiatric symptoms; possible cases of Post-Traumatic Stress Disorder (PTSD); symptoms of panic, generalized anxiety and depression; alcohol dependence and abuse; functioning at home and work; and suicidal ideation. The questionnaire is scored during the same visit, allowing prompt referral for further mental health evaluation and treatment (immediate, if necessary) among those who score above pre-validated thresholds for various mental health conditions, and for those who acknowledge suicidal ideation or substantial disability, irrespective of score. Community members are provided with WTCHP treatment and surveillance, *via* the WTC Environmental Health Center (WTC EHC), if they have physical or mental health symptoms (general health surveillance is not provided). Those that do undergo a standardized protocol that includes a self-accessed exposure history and physical and mental health questionnaires and testing. The exposure history captures information on both acute exposure (from the dust clouds created by the buildings’ collapse) and chronic exposure (from re-suspended dust that had settled in homes, streets and workplaces) [45]. As of 31 March 2014, the WTCHP included 37,281 subjects for the GRC, ~15,000 firefighters and ~5000 survivors [46].

Certification for treatment requires attestation from a WTCHP medical professional that exposures present during the WTC effort (*e.g.*, airborne toxins, heavy lifting or repetitive strains on muscles and joints from work performed on the WTC effort, viewing falling bodies or body parts) played a significant role in aggravating, contributing to, or causing the physical or mental health condition. As of March 31^st^, 2014, 46% (15,133 of the 33,076) of the GRC were certified for at least one WTC-related condition, whereas, by definition, 100% of the over 8600 enrolled community members have a certification [47]. Participants in the WTCHP can also receive Workers’ Compensation and benefits from other disability programs and are eligible to receive compensation for physical harm, including death, from the Victims Compensation Fund, which was included in the federal legislation.

More than 20% of the GRC is suffering persistent physical and mental health problems: the cumulative incidence rates of asthma, sinusitis and abnormal spirometry have been reported to be 27.6, 42.3 and 41.8%, respectively [45], for example. These rates cannot differentiate the diseases diagnosed before 9/11, because of the lack of baseline data, except for the FDNY portion of the responders’ cohort. Multiple comorbidities have been frequently observed: a syndrome of asthma, Gastro Esophageal Reflux Disease (GERD) and sinusitis has been observed in approximately 10% of the GRC. The incidence rate of sarcoid-like granulomatous pulmonary disease has been estimated to be 32 *per* 100,000 person-years, a rate that is elevated compared to published background rates [48]. The cumulative incidences for depression, PTSD, and panic disorder in the non-police responders are 27.5, 31.9 and 21.2%, respectively, and approximately 6% of the cohort suffers from all three [45]. Analyses have shown elevated standardized incidence ratios for all-cancer-sites-combined (SIR = 1.15; 95% CI: 1.06, 1.25), thyroid (SIR = 2.39; 95% CI: 1.70, 3.27), prostate (SIR = 1.21; 95% CI: 1.01, 1.44), soft tissue (SIR = 2.26; 95% CI: 1.13, 4.05), and combined hematopoietic cancer (SIR = 1.36; 95% CI: 1.07, 1.71) [49, 50]. There is also some indication of an elevation in multiple myeloma [51]. In contrast to the GRC (and FDNY), nearly 50% of the patients in the WTC EHC are women (most were local workers). As seen in the GRC, most WTC EHC patients suffer from aerodigestive disorders [52], and rates of screening are positive for PTSD, anxiety and depression. An increasing number of patients are enrolling in the EHC with cancers, including breast, hematopoietic, thyroid and prostate cancer [44]. Incidence rates cannot be determined from the EHC. The WTCHP provides screening for cervical, breast, colon and lung cancer for both GRC and EHC (www.cdc.gov/wtc/cancerfactsheets.html).

#### Fukushima (March 11^th^, 2011 – 3/11 [5])

Two months after the event, the Nuclear Emergency Response Headquarters of Japan published the “Roadmap for Immediate Actions for the Assistance of Nuclear Sufferers” which outlined the need to track the emergency workers long-term, including after retirement, and implement long-term health care activities. In October 2011, the Japanese Ministry of Health, Labour and Welfare (MHLW) established an expert panel on the long-term health care of the workers and published ministerial guidelines [53], based on the Industrial Safety and Health Act, to: (1) establish size-appropriate health management and surveillance at each workplace; (2) conduct annual surveillance, for those who participated in the emergency work, consisting of cancer screening, an eye examination for cataracts with a slit-lamp for those with an effective dose greater than 5 cGy, and thyroid tests for individuals with an effective dose greater than 10 cGy; and (3) provide health guidance to the emergency workers. Employers are required to provide the long-term health care, including health surveillance, for their employees. The government provides the long-term health care for the unemployed, for workers who have changed occupations since the accident, and for those employed by some small-to-medium size companies not directly engaged in radiation work. The MHLW also established a Data Center for the radiation dose and health surveillance data [54].

The MHLW also published an (MHLW-convened) expert panel report which included projections, for emergency workers who worked during the period (March 14 to December 16, 2011) when the effective dose limit was 25 cGy (instead of 10 cGy), that the expected health effects include solid cancers, leukemia, non-cancer diseases, and psychological distress. At the end of 2014, experts in epidemiology, radiation medicine and occupational health were again convened by the MHLW and a cohort study covering all ~20,000 emergency workers was launched [55]. In addition, Fukushima prefecture, is implementing the “Health Survey of Prefectural Residents (Kenmin Kenkou Chosa)” to assess the levels of exposure to radiation and the health status of their residents, with a view to the early detection, prevention and treatment of potential diseases. The baseline survey (Kihon Chosa) was open to everyone residing in the prefecture since 3/11 and estimated individual exposure levels. Data are collected in a database for long-term follow-up, which includes thyroid ultrasound examination for residents who were ≤18 years old on 3/11 and a general health assessment for all residents, regardless of age. Differential leukocyte counts is also obtained for residents of the evacuation areas, as well as examination of mental health status. For pregnant women, specific questionnaires were added to a maternal and child health handbook. Preliminary findings of the surveys [56–58] have not been officially acknowledged by the government at the time of writing.

## Discussion

The information gathered on the disasters and the health programs is summarized in Table 1. As each disaster recovery program varied depending on the antecedent event, the country of origin as well as the resources available, and we could not identify a unique “metric” to identify the effectiveness of these programs. The different aspects were considered in a comparative analysis of the programs and the results show different impacts of each program in the different items. The most updated information located for each program was analyzed also considering the authors’ perspective and consensus, based on their personal qualitative reviewed and direct experience with these disasters.

**Table 1:** Description of the Health Surveillance and Treatment programs of Seveso, Three Mile Island, Bhopal, Chernobyl, WTC and Fukushima

	Seveso, Italy [69]	Three Mile Island (Dauphin County, PA, US) [70, 71]	Bhopal, India [72, 73]	Chernobyl, Ukraine [28, 29]	World Trade Center, US [45, 46]	Fukushima, Japan [74]
Incident date (m/d/y)	7/10/76	3/13/78	12/2/82	4/26/86	9/11/01	3/11/11
Types of exposure (average exposure intensity if known)	2,3,7,8-tetrachlorodibenzo-p-dioxin	Ionizing radiation	Methyl isocyanate gas (MIC) and other unknown chemicals	Ionizing radiation	Cement dust (pH 10.0-11.0) Glass fibers Lead and Heavy Metals Polychlorinated biphenyls (PCBs) Chrysotile Asbestos Toxic Products of Combustion: • Benzene and VOCs • Polycyclic Aromatic Hydrocarbons • Dioxins • Diesel fumes • Sulfur dioxide	Ionizing radiation (<2 mSv – residents) (<680 mSv – workers) Organic and inorganic dust, originating from sludge, rubble and debris, occasionally including asbestos
Estimated exposed workers/responders	37,000 workers and residents	600,000 workers and residents	500,000 workers and residents	600,000	90,000	20,000
Estimated exposed Residents/survivors				5 million	400,000	200,000
Immediate deaths	No immediate deaths	No immediate deaths	Estimated from 3787 to 10,000 (¾ of deaths occurred during the first 72 h of the leak)	29	2753 (2996 including all sites of 9/11 attack)	No immediate deaths
Follow-up cohort	220,000 surveyed	160,000 population within 10 miles of Three Mile Island	80,000, from severely, moderately, and mildly exposed areas and unexposed area controls.	600,000	37,281 workers responders [46] • 48% protective/military service • 23% construction • 7% electrical/telecom repair • 4% transportation • 2% unemployed/retired • 16% other 15,000 Firefighters 5000 resident (‘survivors’)	20,000
Frequency of health surveillance	annual	Permanent TMI Employees as of March 1st, 1979 were surveyed by telephone interview	No formal health surveillance program (Indian Council of Medical Research halted after 1994)	annual	annual	annual
Content of health surveillance	17,000 residents from zones A, B, and R received systematic medical testing through 1984. All school children received skin examinations.	(Elevated cancer rates were reported.)	not applicable	medical examination	Physical Health, Mental Health and Exposure questionnaires, physical examination, pulmonary function test, clinical chemistry. Chest X-Ray every 2 years. Cancer screening for cervical, breast, colon, lung cancer	Eye examination for cataracts (effective dose > 50 mSv) Cancer screening and thyroid tests (effective dose > 100 mSv
Mental health effects	None reported	None reported	Based on reported symptoms: Depression, anxiety, adjustment disorder	Clinical depression, anxiety, PTSD	Major depression, PTSD, panic disorders	Psychological distress, e.g., depression, PTSD
Physical effects	Chloracne [1], peripheral neuropathy and elevated hepatic enzymes	A modest association was found between proximity to TMI and cancer (OR = 1.4; 95% CI = 1.3, 1.6) [71].	Reported pulmonary fibrosis, bronchial asthma, chronic obstructive pulmonary disease (COPD), emphysema, recurrent chest infections, keratopathy and corneal opacities	Cardiovascular diseases	Asthma, sinusitis, abnormal spirometry, gastro-esophageal reflux	to be determined (see text and [75])
Carcino-genic effects	Excess risk of Lymphatic and hematopoietic tissue neoplasms [16] and breast cancer after 20 years follow-up.	High rates of all cancers, lung cancer, and leukemia [71].	Lung, orophrarynx, and oral cavity cancers were reported.	Excess risk of Leukemia, Solid cancers, Thyroid cancers	All cancer sites combined, soft tissue, combined hematopoietic cancers, thyroid, prostate after 10 years follow up.	Too soon for comprehensive cancer risk assessment. At least six workers have exceeded lifetime legal limits for radiation and more than 300 have received significant radiation dose
MeSH term	“Seveso Accidental Release”	“Radioactive Hazard Release”[Mesh] AND (“Pennsylvania/epidemiology”[Mesh] OR “Pennsylvania/statistics and numerical data”[Mesh])	“Bhopal Accidental Release”	“Chernobyl Nuclear Accident”	“September 11 Terrorist Attacks”	“Fukushima Nuclear Accident”
Number of health-related publications through December 2015	21	12	22	1228	957	767

Exposure assessment: although exposure was assessed to some certain extent in Seveso, Chernobyl and Fukushima, none benefited from a timely or systematic strategy, yielding immediate and sequential measurements of physical hazards (chemicals, radioactive, biological, etc.) and psychogenic traumas. Identification of exposed populations: Only in Seveso was the number of exposed individuals known with reasonable certainty, causing an overall underestimate of the impact of these events, especially for the population residing in the vicinities of the events. Implementation of health programs: Health surveillance, treatment and follow-up research were implemented, to varying degrees in Seveso [1], Chernobyl [59–61] and the WTC [46]. They generally focused on the workers and responders, and to a lesser extent on residents and survivors. Exposure-related physical and mental health consequences were identified for the exposed individuals and their progenies, indicating the need for a long-term health care of the affected populations. A health program is planned by the Japanese Ministry of Health Labour and Welfare for the workers exposed to ionizing radiation >10 cGy after the nuclear accidents at Fukushima [53]. No effective program was implemented for Three Mile Island or Bhopal, likely causing further long-term consequences on the exposed populations. Research: Fukushima has generated the largest scientific output so far, followed by the WTCHP and Chernobyl. Benefits and outreach activities: only the WTCHP has implemented systematic programs.

The results of the comparative analysis based on the literature review and the authors’ opinion and experience, lead to specific lessons learned from these disasters.

### Lesson learned and indications for the future

This is the first review of health programs that were instilled in response to large-scale disasters, and that combines information from experts directly engaged in the epidemiological assessments of health outcomes of workers, responders and residential populations at each global site. The following indications are based on the important lessons learned from the described programs.

#### Lesson 1: know who was there

Of primary importance, is to ensure a comprehensive inventory through a tracking system of impacted workers, volunteers and residents immediately after the accident. Access to the disaster site should be diligently restricted to necessary personnel only, to minimize the health impact. This should include the issuance of electronic ID cards with GPS capabilities so that additional information about time and location at the site can be automatically tracked and recorded. Rostering and credentialing recommendation are available from the Emergency Responder Health Monitoring System (www.cdc.gov/niosh/topics/erhms/), which was developed by the National Institute of Occupational Safety and Health in the USA after 9/11.

#### Lesson 2: have public health input to the disaster response

The full spectrum of public health expertise (physicians, epidemiologists, environmental health professionals trained in disaster epidemiology and health consequences of harmful exposures, industrial hygienists, informatics experts) should be involved in a rapid hazard vulnerability assessment of the scope of the disaster and determine public health outcome priorities based on the disaster exposure type(s) – both physical and psychological – and intensity.

#### Lesson 3: collect health and needs data rapidly

Collecting, during the disaster, health and needs information focused on preventing and reducing morbidity and mortality helps to address immediate needs, adjust priorities, and allocate or gather resources. Once the disaster antecedent is known, assessments of evacuation needs, other safety restrictions and contamination considerations for food and water should be made jointly by public health experts, industry, the government, and worker and community organizations [62].

#### Lesson 4: take care of the affected

Monitoring of the disaster’s long-term health effects is necessary. This requires many components to be both put in place and work together: structured data collection (into continually maintained databases) on the physical and mental health outcomes and progression over the lifetime of the affected; detailed exposure assessment (both environmental exposure measurements and biological measurements of the affected populations; adequate physical and mental health treatment and support needs to be provided and accessible; and risk communication must be coordinated between governmental authorities, scientific experts and social scientists. Mental and physical health services should be offered together, preferably in the same healthcare office, healthcare institution or otherwise closely integrated in order to avoid the stigma commonly associated with seeking mental health services [63]. Services needed include referral to specialists for follow-up, group education, support services, and individual counseling [64]. Follow-up of those affected post-disaster enables local public health officials (and public officials) to monitor trends that may necessitate policy changes; evaluate the progress of public health action taken; and identify emerging public health concerns during the recovery or mitigation phase. Research prompted by disasters should be planned according to a decision process based on: i) a clearly defined rationale; ii) availability of baseline data collected before the event [65].

#### Lesson 5: emergency preparedness

In order to prevent and/or minimize the adverse health effects for all affected populations, the full spectrum of public health expertise (examples above) must be involved in hazard vulnerability assessment, disaster planning, and emergency response [62], and the accumulated, long-term data from existing programs – along with input from community leaders, scientists, government officials, and stakeholders – should be used to improve prevention and mitigation strategies for future disasters. Physical and mental health conditions cannot be adequately addressed without making certain that the persons affected have a clean safe place to live in the aftermath of the disaster. The Community Assessment for Public Health Emergency Response (CASPER) toolkit was designed and developed by the CDC to provide rapid, low-cost, household-based information about an affected community’s needs after a disaster, in a helpful format for decision-makers that include personnel from local, state/territorial, regional, or federal public health departments [66]. CASPER has been shown to be a useful tool in disaster situations within the U.S., particularly with regard to the importance of informing communities about available resources, and the provision of services such as debris removal and medications refills [67]. Implementing emergency preparedness can also involve stakeholders (*e.g.*, community groups) that can help to resolve the substantial communication challenges that impeded both preparedness and response. Finally, risk communication would benefit from internationally established, effectively defined and standardized concepts of risk, hazard, vulnerability and disaster.

#### Lesson 6: data driven, needs assessment, advocacy

Data collection is needed in a timely manner; using evidence based and standardized assessment tools. Physical and mental health information and ancillary testing need to be maintained in data base program to yield documentation and comparison to exposure assessment information. Electronic medical record should be used to improve diagnosis and treatment, as well as generating longitudinal reports on health conditions and response to treatment.

All of the above lessons require community support and resources. Credible scientific assessment and advocacy may be able to lessen the complexities of institutional barriers and, where applicable, to integrate disaster risk reduction into strategic reform and program for sustainable development [68]. While early advocacy relies on emotion, compassion, comradery and charity, enduring success will require solid science to document needs and outcomes to validate program investment. Therefore, this type of advocacy requires sound scientific endeavors to collect and analyze requisite data over time on the health effects of the disaster for the community and the responders, suitable to show the effectiveness and efficacy of the health surveillance program, according to a predefined process for decision making [65].

## Conclusion

The human health consequences of natural and/or man-made disasters can be widespread, profound, and long-lasting. Health surveillance and treatment programs are critical for the appropriate management of the health conditions among affected responders and residents in the aftermath of disasters, irrespective of their industrial, natural or terror origin. In parallel with health monitoring and treatment, detailed plans for emergency preparedness must be implemented to prevent or minimize the impact of future threats.
